# Associations between parental bonding during childhood and functional recovery in patients with schizophrenia

**DOI:** 10.1371/journal.pone.0240504

**Published:** 2020-10-15

**Authors:** Junpei Ishii, Fumitoshi Kodaka, Hisatsugu Miyata, Wataru Yamadera, Hikaru Seto, Keisuke Inamura, Hidejiro Higuchi, Yoshiaki Tsuruoka, Masahiro Shigeta

**Affiliations:** 1 Department of Psychiatry, Jikei University Katsushika Medical Center, Katsushika-ku, Tokyo, Japan; 2 Department of Psychiatry, Jikei University School of Medicine, Minato-ku, Tokyo, Japan; 3 Department of Psychiatry, Sobu Hospital, Funabashi-shi, Chiba, Japan; 4 Department of Psychiatry, Otaki Hospital, Isumi-gun, Chiba, Japan; University of Sao Paulo Medical School, BRAZIL

## Abstract

**Introduction:**

Schizophrenia is believed to be etiologically associated with environmental factors. Poor parental bonding, especially arising from “low care” and “overprotection,” may contribute to the prognosis in patients with psychosis. In the present study, we investigated the associations between the aforementioned two different parental bonding types and the prognosis, in terms of the functional recovery, of patients with schizophrenia.

**Methods:**

A total of 89 patients with schizophrenia were recruited, and 79 patients were registered for the study. After the parental bonding types and representative childhood adverse events were assessed, specific items on the PANSS were assessed at 0 and 24 weeks of the study period to define the functional prognosis.

**Results:**

At the end of the 24-week follow-up period, 36% of the patients were judged as showing recovery from schizophrenia. The score for “overprotective attitude,” but not that for “low care,” was found to be significantly higher in the *non-recovery* (defined below) group. Exploratory logistic regression analysis identified only “overprotective attitude” of the parents as being predictive of *non-recovery*. Moreover, a significant negative correlation was found between “low care” and “overprotective attitude” only in the *non-recovery* group.

**Conclusion:**

In the present study, we showed that an overprotective attitude of the parents was associated with *non-recovery* in patients with schizophrenia.

## Introduction

Schizophrenia is believed to be etiologically associated with both genetic and environmental factors. Among the environmental factors that possibly influence the risk of development of schizophrenia are maternal infections in the first trimester [1], urban upbringing [2], migration [3], paternal age [4], cannabis use [5], and childhood adversities [6–10].

From the 1940s to the 1960s, pathological parenting was regarded as a core element in the pathophysiology of schizophrenia. Several theoretical models such as the schizophrenogenic-mother model [11], the fragmented family communications/imbalance model [12, 13], etc., had been proposed. Although these theoretical models contributed to the development of the modern family therapy, several adoption studies suggest that familial psychopathology did not play a role in the pathophysiology of schizophrenia [14, 15].

On the other hand, childhood adversities, including physical/sexual/emotional abuse, neglect, bullying in school and bereavement, can affect the prognosis of patients with schizophrenia. A previous study suggested that patients with psychosis were 2.72 times more likely to have faced adversities in childhood than healthy controls [16]. Moreover, experience of childhood adversity was also found to be positively correlated with the persistence of psychotic symptoms [17].

It has been reported that poor parental bonding, which can be classified into two distinct types, namely, “low care” and “overprotective attitude,” is associated with an earlier age at initial hospitalization and early re-admission in patients with schizophrenia [18]. A low level of attachment to parents has been reported to be associated with a history of childhood abuse, while attachment to close adults is associated with alleviation of trauma-related symptoms [19]. Although the relationship between parental bonding during childhood and psychotic symptoms has been investigated in previous studies, little is known about the relationship between parental bonding and the longitudinal course in patients with schizophrenia.

The longitudinal course in patients with schizophrenia is defined by a combination of social functioning and severity of psychotic symptoms. A previous study suggested the concept of “*recovery*” from schizophrenia, which is defined as a high degree of social adaptation, in addition to remission of clinical symptoms [20]. It has been reported that “*recovery*” is achieved in about 30% of patients with schizophrenia [21, 22].

In the present study, we investigated the relationship between parental bonding during childhood and the longitudinal course, especially “*non-recovery*,” in patients with schizophrenia. First, the participants were interviewed about their experience of adverse events in childhood. Subsequently, the longitudinal prognosis, in terms of “*recovery*” or “*non-recovery*,” was determined after a 24-week follow-up period. Consequently, the associations between “*recovery*”/“*non-recovery*” and the two types of parental bonding during childhood (i.e., “low care” and “overprotective attitude”) were examined.

## Materials and methods

### Study design and patients

The present study was performed as part of a non-interventional, multicenter, prospective research (the Predictors of *Recovery* in Patients with Schizophrenia [PREPS] study). From April 2015 to January 2019, a total of 89 patients with schizophrenia were recruited from the outpatient section of the Department of Psychiatry, the Jikei University Hospital, the Jikei University Katsushika Medical Center, Sobu Hospital, and Otaki Hospital, and 76 of these patients who provided written informed consent were registered in the study. All the patients were diagnosed as suffering from schizophrenia according to the Diagnostic and Statistical Manual of Mental Disorders, Fourth edition, Text Revision (DSM-IV-TR), by five expert psychiatrists (JI, FK, HM, HS, and WY) with at least 5 years’ experience in clinical psychiatry. In our sample size estimation, to examine the influence of parental care and overprotection between the *recovery* and *non-recovery* groups, we referred to a previous study which suggested that 28–36 patients were needed for each group for an alpha of 0.05, beta of 0.2 (i.e., power of 0.8), and an effect size (*d*) of 0.67–0.76 [23]. We also estimated that 20%-40% of the 89 patients (i.e., 18–36) would show recovery from schizophrenia.

The inclusion criteria in the study were: 1) schizophrenia diagnosed according to the DSM-IV-TR, and 2) 20 to 59 years of age. 3) ability to understand the consent process and the questionnaire. The patient’s ability to consent was assessed by the psychiatrist. The exclusion criteria were: 1) presence of severe physical illness, 2) comorbid substance use disorder, and 3) inability to understand enough about the study to provide informed consent.

To investigate the association between parental bonding and the longitudinal course in patients with schizophrenia, a prospective two-wave (0 and 24 weeks) survey was conducted. At 0 week, patients who participated in the study answered a questionnaire on the experience of adversity in childhood and parental bonding (Japanese version of Parental Bonding Instrument [PBI]). These patients were then followed up for 24 weeks, and finally the functional prognosis was classified as “*recovery*” or “*non-recovery*” according to assessment by the Positive and Negative Syndrome Scale (PANSS) and the Global Assessment of Functioning (GAF) scale at 0 and 24 weeks.

As for the validity of the PBI, the criterion-related validity, the stability of the scores over a 20 year-period and the effects of psychiatric symptoms have been confirmed by previous studies. In a study conducted in healthy college students, the scores on the PBI in the students were correlated with those of theirs mothers [24]. Another study performed in a healthy population and patients with psychiatric disorders, the scores for the PBI showed no significant changes over a 20 year-period [25]. Another study performed in patients with schizophrenia showed a strong correlation between the scores on the PBI in patients who remained in a disturbed state versus those who showed an improvement of the state [26].

The present study was conducted with the approval of the Ethical Committee of the Jikei University School of Medicine (No. 7493), in compliance with the principles laid down in the Helsinki Declaration of Human Rights.

### Clinical assessment

#### Assessment of childhood adverse events and parental bonding

To investigate parental bonding and experience of adverse events during childhood, we conducted an assessment to determine the parental “low care” or “overprotective attitude,” any experience of bereavement of first- or higher-degree relatives, and any experience of having been bullied in school (6–12 years old).

Parental bonding during childhood was assessed by the Japanese version of the PBI [27, 28]. The PBI consists of two categories, i.e., “parental care” and “overprotection,” and 25 items, including 12 items pertaining to “care” and 13 items pertaining to “overprotection.” Examples of items pertaining to “care” are “Spoke to me with a warm and friendly voice,” and “Appeared to understand my problems and worries”; examples of items pertaining to “overprotection” are “Did not want me to grow up,” and “Tried to control everything I did.” The total score on the “care” scale (PBI-Care) ranged from 0 to 36, while that on the “overprotection” scale (PBI-Overprotection) ranged from 0 to 39. These categories were independent of each other [27].

#### Assessment of the functional prognosis

To assess the functional prognosis, we defined “*recovery*” and “*non-recovery*,” according to the definitions provided in previous studies [20, 29]. We classified the participants into two groups, namely the “*recovery*” group and “*non-recovery*” group according to the scores on the 8 PANSS subscales (delusions (P1), disorganization (P2), hallucinatory behavior (P3), blunted affect (N1), social withdrawal (N4), lack of spontaneity (N6), mannerism (G5), and unusual thought content (G9)) and the GAF score at 24 weeks. The GAF scale measures the degree of mental illness by rating psychological, social and occupational functioning. We classified patients with “mild” or low scores (3 points or lower) in all of P1, P2, P3, N1, N4, N6, G5 and G9, and a GAF score of 81 or more at 0 and 24 weeks into the “*recovery*” group, and the others into the “*non-recovery*” group [20, 29].

### Statistical analysis

All data analyses were performed with SPSS version 21.0 (IBM, Chicago, IL). The statistical significance level was set at *p* <0.05.

#### Comparison of experience of adverse events and parental bonding during childhood between the *recovery* and the *non-recovery* groups

To examine childhood experience of adverse events and parental bonding in the *recovery* group, we compared the experience of having been bullied, experience of bereavement, and scores on PBI-Care and PBI-Overprotection in the subjects. To adjust for the effects of positive symptoms, we used analysis of covariance (ANCOVA) adjusted for the scores for the positive symptoms on PANSS. For the categorical variables (experience of having been bullied and experience of bereavement), a chi-squared test was performed, while a t-test was performed for comparing continuous variables (PBI-Care and PBI-Overprotection).

#### Associations of different parental bonding types during childhood with the prognosis

To examine the relationships between different parental bonding types during childhood and the functional prognosis in patients with schizophrenia, exploratory logistic regression analysis was performed using the parental bonding types and representative childhood adverse events. Scores on PBI-Care and PBI-Overprotection, and experience of bullying and bereavement in school age were adopted as the independent variables. The functional prognosis after 24 weeks (i.e., *recovery* and *non-recovery*) was used as the dependent variable.

#### Relationship to parental “care” and “overprotection” in the *recovery* and the *non-recovery* groups

To estimate the influence of the parental bonding type during childhood in the *recovery* group and *non-recovery* group, we performed *post-hoc* correlation analysis between the scores on the PBI-Care and PBI-Overprotection for each group. Pearson’s product-moment correlation coefficients (*r*) were used to examine the correlation between PBI-Care and PBI-Overprotection.

## Results

**Fig 1:**
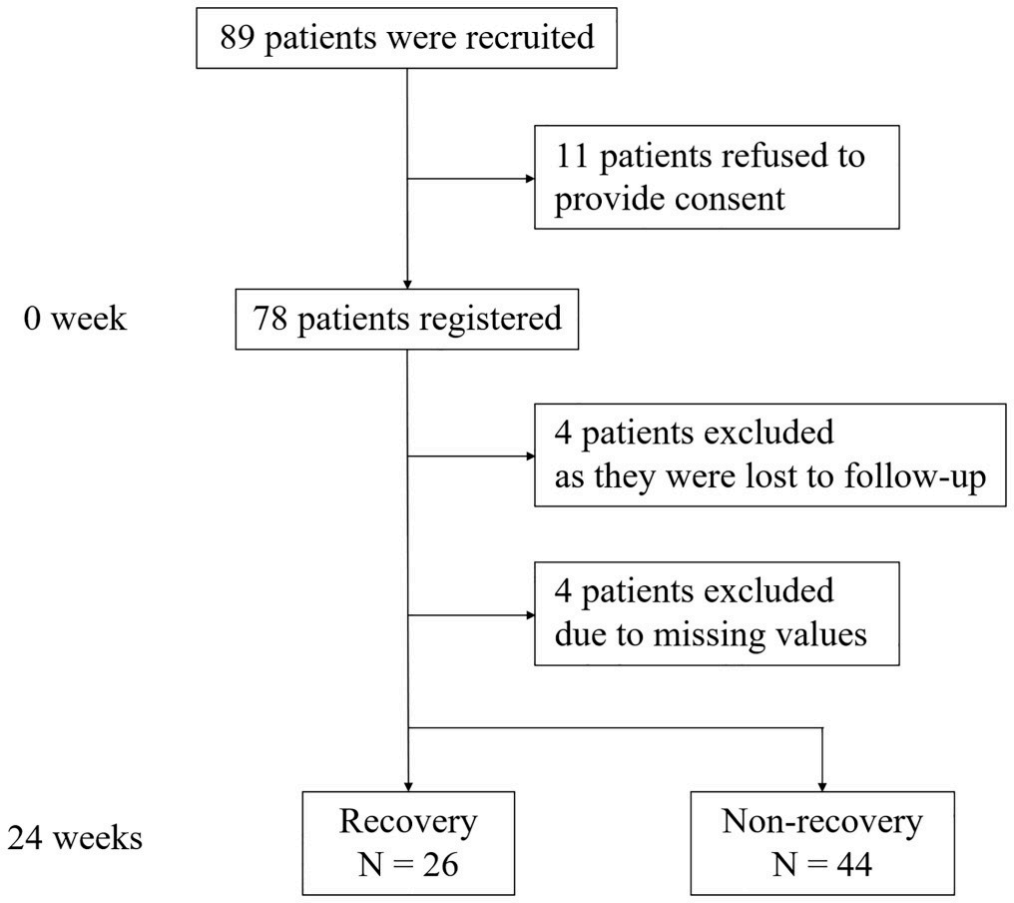
Consort diagram.

Of the 89 patients recruited for the study, 78 who provided informed consent for participation were enrolled in the study. Finally, the data of 70 patients were analyzed, after eight patients withdrew their consent or there were missing values. After 24 weeks, 26 patients with schizophrenia were categorized into the *recovery* group, while 44 patients were classified into the *non-recovery* group (Fig 1).

The PANSS scores at the baseline, the chlorpromazine-equivalent dose (CED) and the duration of untreated psychosis (DUP) were significantly higher in the *non-recovery* group than in the *recovery* group (Table 1). The PANSS score at the baseline was 34.7 ± 3.9 in the *recovery* group and 50.9 ± 12.7 in the *non-recovery* group. The CED was 346.0 ± 292.1 in the *recovery* group and 866.0 ± 600.4 in the *non-recovery* group. The DUP was 5.7 ± 7.3 in the *recovery* group and 17.8 ± 23.5 in the *non-recovery* group. The GAF score at 24 weeks was 84.5 ± 3.3 in the recovery group and 61.5 ± 10.2 in the *non-recovery* group.

**Table 1:** Comparison of the demographic data between the *non-recovery* and *recovery* groups.

	*Non-recovery* group (N = 44) (mean ± s.d.)	*Recovery* group (N = 26) (mean ± s.d.)	*p*-value
Age	40.1 ± 9.4	40.2 ± 8.7	0.97
Gender (% male)	22 (59.3)	12 (46.2)	0.81
PANSS total (week 0)	50.9 ± 12.7	34.7 ± 3.9	<0.01
PANSS 8 (week 0)	16.2 ± 5.3	9.6 ± 1.8	<0.01
PANSS 8 (week 24)	16.4 ± 5.7	9.2 ± 1.2	<0.01
GAF (week 0)	61.3 ± 10.4	82.8 ± 5.6	<0.01
GAF (week 24)	61.5 ± 10.2	84.5 ± 3.3	<0.01
CED (mg)	866.0 ± 600.4	346.0 ± 292.1	<0.01
DUP (months))	17.8 ± 23.5 (N = 33)	5.7 ± 7.3 (N = 24)	0.01
Onset age	24.7 ± 6.5 (N = 40)	26.8 ± 7.9	0.24

s.d., standard deviation; PANSS, Positive and Negative Syndrome Scale; GAF, Global Assessment of Functioning scale; CED, chlorpromazine-equivalent dose; DUP, duration of untreated psychosis.

In regard to the possible influence of the parental bonding type, only the score for “overprotective attitude” was significantly higher in the *non-recovery* group than in the *recovery* group (Table 2). The scores for PBI-Overprotection in the *non-recovery* and *recovery* group were 15.4 ± 7.4 and 11.3 ± 4.6, respectively (*p* = 0.01). In contrast, the scores for PBI-Care in the *non-recovery* group was lower than that in the *recovery* group (22.1 ± 7.4 and 23.2 ± 4.2, respectively), although the difference was not statistically significant (*p* = 0.44).

**Table 2:** Comparison of the experience of adverse events and parental bonding type during childhood between the *recovery* and *non-recovery* groups.

	*Non-recovery* group (N = 44) (mean ± s.d.)	*Recovery* group (N = 26) (mean ± s.d.)	*p*-value
Bullying (%)	18 (40.9)	7 (26.9)	0.02*
Bereavement (%)	11 (25.0)	3 (11.5)	<0.01*
PBI-C	22.1 ± 7.4	23.2 ± 4.2	0.44
PBI-O	15.4 ± 7.4	11.3 ± 4.6	0.01*

PBI, Parental Bonding Instrument; PBI-C, care; PBI-O, overprotection

There were significant differences in the experience of having been bullied and experience of bereavement between the *non-recovery* and *recovery* groups. The number of patients with the experience of having been bullied in the *non-recovery* group was significantly higher than that in *recovery* group (40.9% vs. 26.9%) (*p* = 0.02). The number of the patients with the experience of bereavement was also significantly higher in the *non-recovery* group as compared to the *recovery* group (25.0% vs. 11.5%) (*p* = 0.01).

Of the independent variables, an overprotective attitude of the parents was identified as the only variable that was strongly predictive of *non-recovery* (β = 0.30, *t* = 2.30, *p* = 0.02) (Table 3).

**Table 3:** Associations of different parental bonding types during childhood and the prognosis in schizophrenia patients.

	β	*t*-value	*p*-value
Bullying	0.12	1.01	0.32
Bereavement	0.13	1.12	0.27
PBI-C	0.03	0.21	0.83
PBI-O	0.30	2.31	0.02*

PBI, Parental Bonding Instrument; PBI-C, care; PBI-O, overprotection

**Fig 2:**
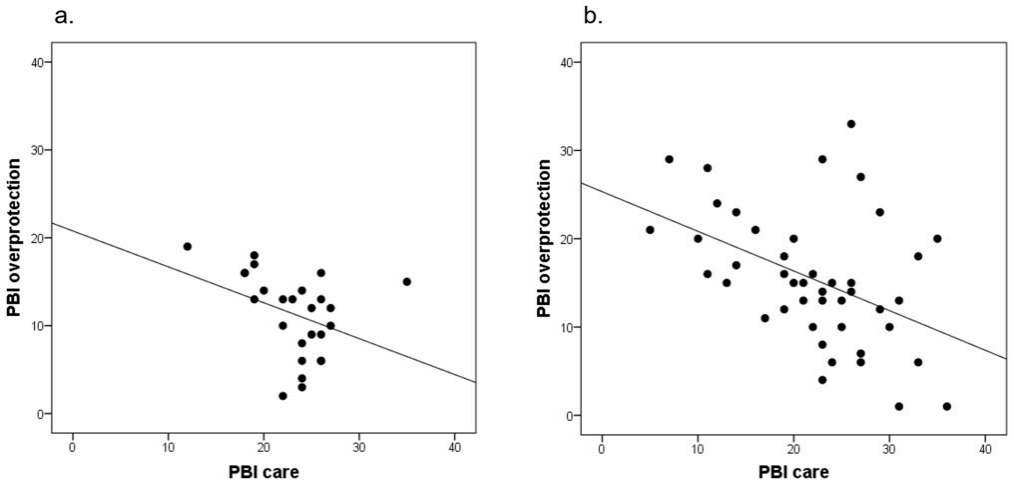
Relationships between parental “low care” and “overprotective attitude” during childhood in the *recovery* (a) and *non-recovery* (b) groups.

A negative correlation was observed between the score for PBI-Care and the score for PBI-Overprotection in the *non-recovery* group (*r* = -0.45, *p* = 0.02), whereas, no significant correlation between the two was observed in the *recovery* group (*r* = -0.37, *p* = 0.06) (Fig 2).

## Discussion

In the present study, we showed that an overprotective attitude of the parents was associated with *non-recovery* from schizophrenia.

### Comparison of the experience of adverse events and parental bonding during childhood between the recovery and non-recovery groups

The score for PBI-Overprotection was significantly higher in the *non-recovery* group than that in the *recovery* group, indicating that an “overprotective attitude” of the parents was more frequently encountered in the patients of the *non-recovery* group. These findings are consistent with previous reports of a strong correlation between the parents’ high expressed emotion (EE) and disease relapse in patients with schizophrenia [30, 31]. Varese et al. reported that emotional abuse was associated with a higher risk of development of psychosis than physical/sexual abuse and neglect [16].

In contrast, the score for PBI-Care in the *non-recovery* group was lower than that in the *recovery* group, even though the difference was not statistically significant. This indicates that the association between a “low care” attitude of the parents and the prognosis of patients with schizophrenia was rather weak. Parker et al. reported that parental “low care” and “overprotection” were associated with a younger age at initial hospitalization and re-admission. Our results differed slightly from those of the previous study, in that the age at initial hospitalization did not differ significantly between the *non-recovery* and *recovery* groups.

The combination of “low care” and “overprotective attitude” could be associated with *non-recovery* from schizophrenia. In a previous study, the mean scores for “care” and “overprotection” were 24.9 and 13.3, respectively, in the general population [27]. In our present study, the mean scores for “care” in both groups were lower than those reported in a previous study (23.2 in the *recovery* group, 22.1 in the *non-recovery* group), showing that patients with schizophrenia can often receive “low care” from their parents. On the other hand, the mean score for “overprotection” in the *recovery* group was lower (11.3), while that in the *non-recovery* group was higher (15.4) than the corresponding values reported in a previous study, indicating that an “overprotective attitude” of the parents during childhood could have an important influence on the functional recovery. Parker et al. described “low care” and an “overprotective” parenting style as “affectionless control”. In a previous study, an affectionless control parenting style was demonstrated to be associated with psychotic symptoms and trauma in patients presenting with their first psychotic episode and patients with borderline personality disorders [32].

Significant differences in the experience of having been bullied and experience of bereavement were observed between the groups. Varese et al. report that such experiences were associated with the development of psychosis. These findings indicate that these events may not only be associated with the development of psychosis, but also with the prognosis of patients with schizophrenia.

### Associations of different parental bonding types during childhood and the prognosis of schizophrenia patients

Our exploratory logistic regression analysis identified PBI-Overprotection score as being the most strongly predictive of the prognosis in patients with schizophrenia (β = 0.30, *t* = 2.30, *p* = 0.02), indicating that a high score for PBI-Overprotection could be predictive of *non-recovery from* schizophrenia. Butzlaff et al. estimated that the mean effect size for EE predicting relapse was 0.30 [31]. Although there have been few previous studies on the relation between an overprotective attitude of the parents and the prognosis, an overprotective attitude of the parents during the patient’s childhood appears to be a risk factor for both relapse and a poor prognosis.

### Relationship between the parental “low care” and “overprotective attitude” during childhood in the recovery and non-recovery groups

Our *post-hoc* correlation analysis revealed a significant negative correlation between the score for PBI-Care and the score for PBI-Overprotection in the *non-recovery* group (*r* = -0.45, *p* = 0.02), but not the *recovery* group (*r* = -0.37, *p* = 0.62). As Parker et al. suggested that the scores for PBI-Care and PBI-Overprotection were independent of each other [27], the presence of a significant negative correlation between the PBI-Care and PBI-Overprotection scores in the *non-recovery* group alone indicates the specific attitudes of the parents of the patients in the *non-recovery* group.

The specific combination of “low care” and “overprotective attitude” of the parents in the patients of the *non-recovery* group could be derived from the patients’ vulnerability and poor social adjustment during childhood. Bebbington et al. reported that patients with schizophrenia spectrum disorders could be more vulnerable to the impact of adverse events in childhood because of their genetic predisposition [30]. Done et al. reported that children who later developed schizophrenia showed a greater degree of social maladjustment, especially in relation to hyperreactive behaviors, at the age of 7 years than controls [33].

### Study limitations

There were several limitations of the current study. First of all, all the data were obtained from the patients, but not the parents. Therefore, the data could be biased because of potential cognitive dysfunction and/or psychotic symptoms in the patients with schizophrenia. In the statistical analysis conducted by ANCOVA, although we applied the PANSS positive scores as a covariate to examine the difference between the PBI scores in the *recovery* and *non-recovery* groups, it is possible that the results were affected by the presence of psychiatric symptoms such as idea of persecution.

To estimate the sample size for our study, we referred to a previous study on the parent-child relationships between patients with depressive disorders and healthy controls using the PBI [23]. In this study, the effect sizes were 0.67 (maternal care) and 0.76 (overprotection). Therefore, when the alpha was set at under 0.05 and beta at 0.2 (i.e., to obtain a statistical power of 0.8), the estimated sample size was 36 for maternal care and 28 for overprotection. Although we estimated a recovery rate of 20%-40% in our 89 patients with schizophrenia (i.e., 18–36), there were 26 patients in the *recovery* group and 44 patients in the *non-recovery* group. Because the number of participants was rather small, especially to examine the effect of maternal care between the *recovery* and *non-recovery* groups, the alpha error for maternal care could have occurred in the present study.

The study also has the limitation on the representativeness. Although we designed the study as a multicenter research project to allow our results to be generalized, our results could have been affected by selection bias due to the rather small number of institutes located in the biased area.

As for the concept of recovery from schizophrenia, we followed the concept adopted in a previous study [29], in which recovery was defined based on a combination of the scores on the PANSS and GAF, in terms of feasibility. Recovery from schizophrenia as originally proposed by Robert Paul Liberman [20] is defined based on a multi-dimensional concept of low score on the Brief Psychiatric Rating Scale (BPRS), 50% of time of employment, independent management of daily activities, and participation in social or recreational activities at least once in a week. In a future study, we plan to adopt this multi-dimensional concept of recovery.

There could be some latent mediators on social isolation that accounted, at least in part, for the relationship between the parental bonding style and likelihood of recovery from schizophrenia observed in our study. A previous study on overparenting conducted in parents and their children who had already grown to become adults suggested that overparenting was associated with ineffective coping skills in the children, such as blaming themselves and distancing [34]. Such ineffective coping skills could lead to the isolation of the children from society. Development of schizophrenia in socially isolated persons could be associated with a worse prognosis, due to the potentially long DUP [35].

## Conclusion

In the present study, we showed that an overprotective attitude of the parents was associated with *non-recovery* in patients with schizophrenia.

## Supporting information

S1 Data (CSV)
